# A simple technique for suturing inside the nose

**DOI:** 10.1308/003588412X13373405386015e

**Published:** 2012-09

**Authors:** SEJ Farmer, C Roberts

**Affiliations:** Abertawe Bro Morgannwg University Health Board,UK

**Figure 1:**
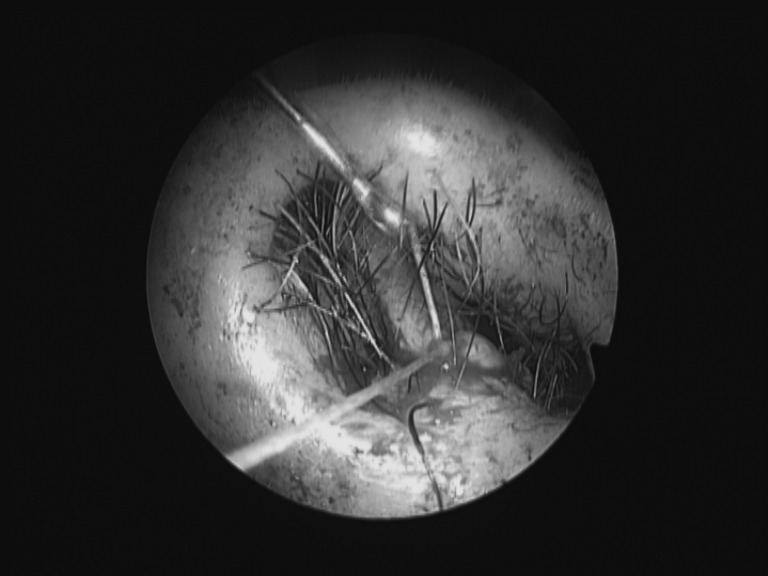
Tying in the nose using a Jobson Horne probe

Suturing incisions deep inside the nose can be difficult and knot tying risks ‘cheese wiring’ the skin of the nasal vestibule. We have found using a Jobson Horne ring probe useful (Fig 1). This instrument is usually readily available and more commonly used for cerumen removal. It can be threaded over one end of the suture after each throw and passed inside the nose to provide counter tension, advancing each throw of the knot without damaging the nasal skin.

